# A Discrete Informational Framework for Classical Gravity: Ledger Foundations and Galaxy Rotation Curve Constraints

**DOI:** 10.3390/e28040477

**Published:** 2026-04-20

**Authors:** Megan Simons, Elshad Allahyarov, Jonathan Washburn

**Affiliations:** 1 Recognition Physics Institute, Austin, TX 78701, USA; exa54@case.edu (E.A.); jon@recognitionphysics.org (J.W.); 2 Institut für Theoretische Physik II: Weiche Materie, Heinrich-Heine Universität Düsseldorf, Universitätsstrasse 1, 40225 Düsseldorf, Germany; 3 Theoretical Department, Joint Institute for High Temperatures, Russian Academy of Sciences (IVTAN), 13/19 Izhorskaya Street, Moscow 125412, Russia; 4 Department of Physics, Case Western Reserve University, Cleveland, OH 44106, USA

**Keywords:** discrete exterior calculus, weak-field gravity, Poisson equation, fractional calculus, nonlocal response, scale-free kernel, linear response theory, galaxy rotation curves, SPARC, modified gravity, phenomenological effective field theory, informational constraints, golden ratio

## Abstract

The weak-field, quasi-static regime of gravity is commonly described by the Newton–Poisson equation as an effective response law. We construct this response within a cost-first discrete variational framework. The Recognition Composition Law (RCL) uniquely selects a reciprocal closure cost within the restricted quadratic symmetric composition class; together with the discrete ledger axioms AX1–AX5 (including conservation) and standard DEC refinement, the Newton–Poisson baseline is then recovered in the instantaneous-closure limit. Conditional on Assumption AS1 (scale-free latency) and Assumption AS2 (causal frequency–wavenumber ansatz), allowing finite equilibration introduces fractional memory into the response, yielding a scale-free modification of the source–potential relation characterized by a power-law kernel $w_{\ker}\left(k\right)=1+C{\left(k_{0}/k\right)}^{\alpha }$ in Fourier space. The kernel exponent $\alpha =\frac{1}{2}\left(1-\varphi^{-1}\right)\approx 0.191$, where $\varphi =(1+\sqrt{5})/2$, is derived from self-similarity of the discrete ledger closure; the amplitude $C=\varphi^{-2}\approx 0.382$ is identified as a hypothesis from a three-channel factorization argument. We evaluate this quasi-static kernel-motivated response against SPARC galaxy rotation curves under a strict global-only protocol (fixed M/L=1, no per-galaxy tuning, conservative $\sigma_{\mathrm{tot}}$), using a controlled multiplicative surrogate for the full nonlocal disk operator implied by the kernel. In this deliberately over-constrained setting, the surrogate interface achieves $\mathrm{median}(\chi^{2}/N)=3.06$ over 147 galaxies (2933 points), outperforming a strict global-only NFW benchmark and remaining less efficient than MOND under identical constraints. The analysis is restricted to the non-relativistic, quasi-static sector and should be read as a falsifier-oriented galactic-regime consistency check of the scaling window, not as a relativistic completion or a claim of Solar System viability without additional UV regularization/screening.

## 1. Introduction

The weak-field, quasi-static regime of gravity is described operationally by a scalar response law relating matter sources to a potential: the Newton–Poisson equation. This description is understood as an effective limit rather than a fundamental statement about gravity [1,2]. Observations of galaxy rotation curves [3], gravitational lensing [4], and cosmic microwave background anisotropies [5,6] reveal systematic departures from baryonic Newtonian predictions. Within the standard ΛCDM framework, these are explained by invoking cold dark matter halos with complex per-galaxy profiles [7,8,9]. While cosmological simulations successfully reproduce large-scale structure [10,11], persistent small-scale tensions remain: the missing satellites problem [12], the too-big-to-fail problem [13], and cusp–core discrepancies [14]. These small-scale tensions motivate both refinements of dark matter models and modifications to the gravitational response law.

Phenomenological alternatives modify the gravitational response law directly. Modified Newtonian Dynamics (MOND) [15] introduces an acceleration scale $a_{0}≃1.2\times 10^{-10}$ m/s^2^ below which dynamics deviate from Newtonian predictions, fitting galaxy rotation curves with a single global parameter [16,17]. The empirical Radial Acceleration Relation (RAR) [17,18] demonstrates tight correlation between observed and baryonic accelerations across diverse galaxy populations, suggesting a universal modification mechanism. However, MOND struggles with cluster dynamics [19] and lensing observations [4] and lacks a fully predictive covariant completion, though relativistic extensions have been proposed [16]. Broader classes of modified gravity theories—f(R) gravity, scalar–tensor theories, and nonlocal models [20,21,22,23]—have been extensively explored as alternatives to dark matter, though many face stringent observational constraints from Solar System tests [24], gravitational wave observations [25], and cosmological probes.

Complementary to continuum field theories, discrete approaches to gravity have a long history. Regge calculus [26] discretizes spacetime geometry on simplicial complexes, enabling numerical general relativity. Loop quantum gravity [27,28,29] quantizes geometry using spin networks, yielding discrete spatial structures at the Planck scale. Causal set theory [30,31,32] posits spacetime as fundamentally discrete and partially ordered. Causal dynamical triangulations [33,34] employ path-integral quantization on dynamically assembled simplicial lattices. Discrete exterior calculus (DEC) [35,36,37] provides a variational framework for field theories on cellular complexes, preserving topological and conservation properties in the discrete setting. In parallel, information-theoretic proposals suggest that gravity emerges from thermodynamic or entropic principles: Jacobson’s thermodynamic derivation of Einstein’s equations [38], holographic entropy bounds [39,40], and Verlinde’s entropic gravity [41,42] reinterpret gravitational dynamics as emergent from microscopic information content [43]. Padmanabhan’s work [44] connects spacetime thermodynamics to cosmological horizons. The discrete gravity approaches (Regge calculus, LQG, causal sets) target Planck-scale or cosmological regimes and do not, by themselves, yield predictions in the quasi-static weak-field sector. The information-theoretic proposals (Jacobson, Verlinde, Padmanabhan) remain phenomenological at the Newtonian level, lacking unique falsifiable predictions in the regime where the present work operates.

High-quality rotation-curve data from SPARC [45] and related surveys provide stringent tests for modified gravity theories. Studies fitting SPARC galaxies with MOND consistently achieve $\chi^{2}/N$∼1–2 per galaxy [18,46], demonstrating MOND’s empirical efficiency. Dark matter halo models with per-galaxy freedom (NFW profiles with varying mass and concentration) similarly achieve excellent fits [47] but require tuning multiple parameters per object. Recent work emphasizes the importance of global-only protocols—constraining entire galaxy samples with shared parameters—to fairly compare predictive power across frameworks [17,18]. Under strict global-only constraints (fixed stellar mass-to-light ratios, no per-galaxy halo adjustments), ΛCDM performs poorly compared to MOND’s single-parameter global fit. This establishes globality as a critical empirical benchmark.

We develop the discrete informational framework (DIF), a cost-first variational construction for the weak-field quasi-static sector. Imposing the Recognition Composition Law (RCL) uniquely determines a reciprocal closure cost J(x) (Section 2). Together with structural ledger axioms AX1–AX5 (Section 3), this provides the discrete scaffold for a DEC refinement limit that recovers the Newton–Poisson baseline (Section 4). Conditional on Assumption AS1 (scale-free latency) and Assumption AS2 (causal frequency-to-wavenumber mapping), finite equilibration introduces fractional memory into the source–potential relation and yields, in the quasi-static sector, a Fourier-space source-side kernel $w_{ker}\left(k\right)=1+C{\left(k_{0}/k\right)}^{\alpha }$ (Section 5). Within the specific self-similar closure recursion adopted here, the kernel exponent $\alpha =\frac{1}{2}\left(1-\phi^{-1}\right)\approx 0.191$ is structurally selected (Section 7.1), while the amplitude $C=\phi^{-2}\approx 0.382$ is retained only as a labeled hypothesis from a three-channel factorization argument (Section 7.2).

We evaluate the kernel-motivated response against SPARC galaxy rotation curves [45] under a deliberately strict global-only protocol (Section 6): 147 galaxies (2933 points), globally shared surrogate parameters $\left(A,\alpha ,r_{0}\right)$, fixed M/L=1, and a conservative total uncertainty model (Appendix D). The forward model is a controlled multiplicative surrogate for the full nonlocal disk operator, adopted here to test whether a single global scale-free enhancement can survive a deliberately strict no-per-galaxy-tuning benchmark. Accordingly, absence of falsification under these intentionally over-constrained conditions establishes viability in the galactic scaling window, not confirmation, uniqueness, relativistic completeness, or Solar System viability.

The remainder of this paper is organized as follows. Section 2 introduces the Recognition Composition Law and derives the canonical closure cost J(x). Section 3 states the structural ledger axioms (AX1–AX5) and records the resulting discreteness, conservation, and exactness properties used in the DEC bridge. Section 4 proves that instantaneous closure recovers Poisson (Theorem 1). Section 5 introduces finite latency and shows how the phenomenological assumptions AS1–AS2 define the fractional kernel within this effective framework (Derived Result 1). Section 6 presents the SPARC empirical test. Section 7 derives the conditional theory-target exponent α from self-similar closure and identifies the amplitude *C* as a labeled hypothesis from a three-channel factorization argument. Section 8 collects limitations and falsification routes. Section 9 summarizes results and discusses future tests. Appendix A, Appendix B, Appendix C, Appendix D, Appendix E and Appendix F provide proof and computational details; Appendix E documents exploratory sensitivity of the surrogate parameters to objective choice.

## 2. Cost-First Foundations and the Recognition Composition Law

**Figure 1:**
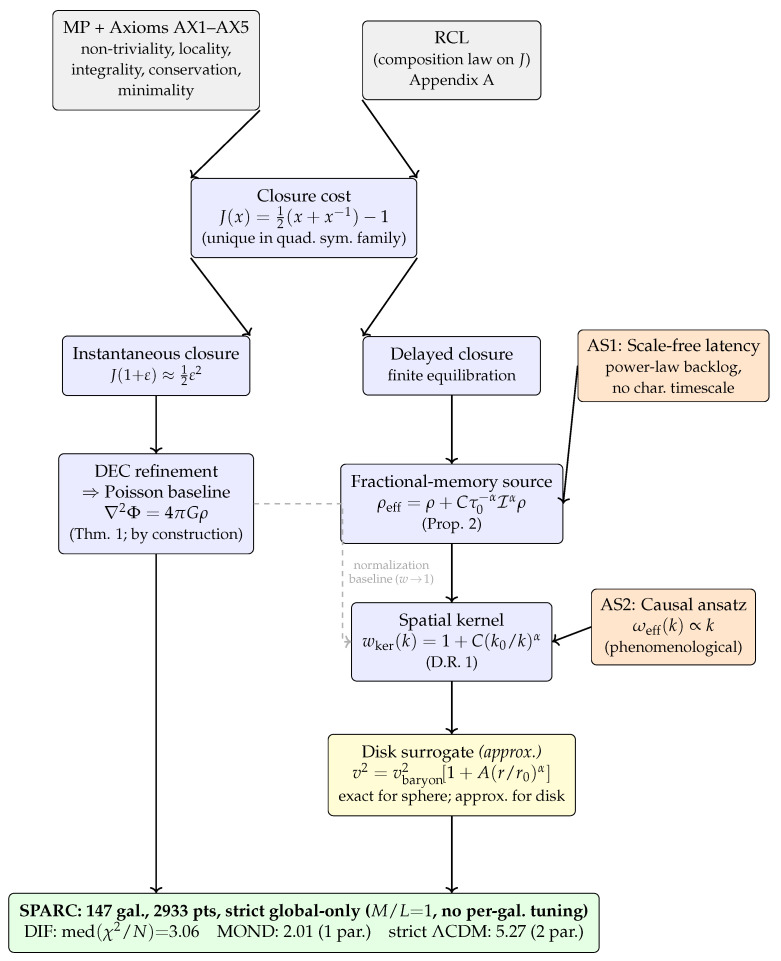
High-level schematic of the informational framework: the Recognition Composition Law uniquely fixes the cost structure, and together with MP and axioms AX1–AX5, it specifies the discrete ledger scaffold. Normalization in the refinement limit recovers the Poisson baseline, while finite equilibration introduces a scale-free kernel constrained by derived parameters. *Color key*—grey: structural inputs (MP, axioms AX1–AX5, composition law RCL); blue: derived mathematical consequences; orange: phenomenological assumptions (AS1, AS2; not derived from first principles); yellow: practical surrogate replacing the exact nonlocal disk convolution; green: empirical test. MP+AX1–AX5 and RCL are co-equal independent inputs converging on J(x); AS1 (scale-free latency) and AS2 (causal $\omega_{\mathrm{eff}}\propto k$) enter as separate side inputs at their respective logical steps; the disk surrogate is an explicit approximate step between $w_{\ker}\left(k\right)$ and the SPARC test.

This section states the Recognition Composition Law (RCL) and the reciprocal closure cost it fixes, establishing the cost-first starting point of the framework. The dependency chain from structural inputs (MP, AX1–AX5, RCL) to the Poisson baseline, the AS1–AS2 conditional kernel, and the strict global-only SPARC test are summarized in Figure 1; the framed status summary below highlights the epistemic status of each ingredient.

For interpretation, we summarize the assumptions and epistemic status:

(i) The Poisson baseline is by construction: the discrete ledger axioms and Recognition Composition Law (RCL) are chosen so that the instantaneous-closure refinement limit reproduces Newtonian gravity (Section 3 and Section 4).
(ii) The primary phenomenological assumptions are AS1 (scale-free equilibration latency) and AS2 (a frequency-to-wavenumber mapping $\omega_{\mathrm{eff}}\left(k\right)\propto k$), which connect temporal causal response to spatial Fourier modes and generate the kernel $w_{\ker}\left(k\right)$ (Section 5).
(iii) The analysis is non-relativistic, quasi-static, and linear-responsive. No claims are made here about lensing, cosmology, gravitational waves, or post-Newtonian tests without an explicit covariant/dynamical completion.
(iv) The galaxy interface is a surrogate: the rotation-curve model uses a controlled multiplicative closure ansatz as a surrogate for the full nonlocal disk convolution implied by $w_{\ker}\left(k\right)$ (Section 6.1.2).
(v) The rotation-curve result is a consistency check: we enforce a strict global-only protocol (fixed M/L=1, no per-galaxy tuning) and a conservative total error model $\sigma_{\mathrm{tot}}$ (Appendix D). The absence of falsification is not confirmation of the framework.

### 2.1. The J(·) Cost Formalism

RCL fixes a unique reciprocal cost (Appendix A); only its near-equilibrium quadratic form $J\left(1+\varepsilon \right)\approx \frac{1}{2}\varepsilon^{2}$ is needed for the DEC bridge to the Poisson baseline (Proposition 1 below).

Positive ratios x>0 parametrize mismatch relative to equilibrium, with the reciprocal cost J:(0,∞)→R vanishing at x=1. In the gravity application, *x* is an auxiliary mismatch variable in an effective discrete constraint model: J(x)=0 denotes local closure (x=1), and J(x)>0 denotes an unclosed imbalance; delayed closure corresponds to persistence of nonzero *J*-cost contributions.

### 2.2. Axiomatic Origin: RCL and the Reciprocal Cost Functional

**Proposition** **1** (Reciprocal closure cost (from RCL [48,49]; proved in Appendix A))**.** *Under RCL together with standard regularity conditions for a non-constant J, and imposing $J^{\prime \prime }\left(1\right)=1$, the reciprocal closure cost is uniquely selected within the restricted quadratic symmetric composition class as*

(1) $$J\left(x\right)=\frac{1}{2}\left(x+x^{-1}\right)-1,$$

*and near equilibrium x=1+ε, one has*

(2) $$J\left(1+\varepsilon \right)=\frac{1}{2}\;\varepsilon^{2}+O\left(\varepsilon^{3}\right),$$

*which is the only property of J needed to recover the Dirichlet energy/Poisson limit in Section 4.*

**Figure 2:**
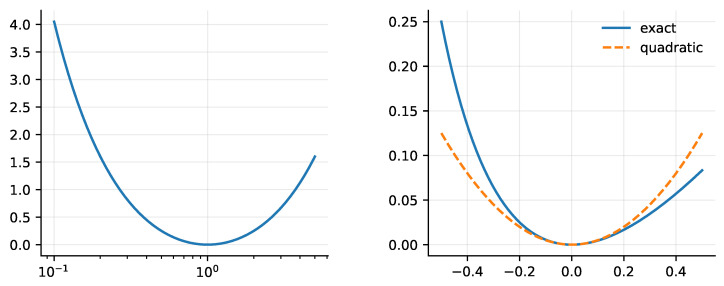
Reciprocal cost J(x) and its near-equilibrium limit: $J\left(x\right)=\frac{1}{2}\left(x+x^{-1}\right)-1$ is reciprocal with a unique minimum at x=1, and $J\left(1+\varepsilon \right)\approx \varepsilon^{2}/2$ yields the Dirichlet energy (Poisson) baseline in the refinement limit.

Figure 2 illustrates the global reciprocity symmetry and the near-equilibrium quadratic regime used in the continuum bridge.

## 3. Emergence of the Discrete Informational Ledger

The discrete ledger structure is the scaffold on which the RCL-selected cost J(x) acts: given axioms AX1–AX5 (Appendix B) and J(x) from Section 2, cost minimization implies discreteness, double-entry conservation, and (under closed-chain neutrality) exactness. This section outlines these structural consequences at the level needed for the DEC bridge.

### 3.1. Discreteness and Conservation from Cost Structure

The reciprocal cost *J* has a unique local minimum at x=1 with positive curvature ($J^{\prime \prime }\left(1\right)=1$); so in a purely continuous configuration space, one can generally move along sufficiently small directions with arbitrarily small incremental costs. This observation does not deny that continuous variational problems can have strict minimizers; rather, it highlights a distinct requirement of the present ledger interpretation: the existence of robust, distinguishable ledger states separated by finite cost barriers (“lock-in”) so that recognition events can be recorded as discrete transactions. In this sense, an additional structure—implemented here as a discrete cellular complex supporting integer cochains—provides a natural mechanism for stable, isolated ledger states. Formal conditions under which a discrete state structure is enforced or selected by the *J*-cost (beyond the present motivation) are discussed in the companion framework exposition [49].

The reciprocity symmetry of the cost function, $J\left(x\right)=J\left(x^{-1}\right)$, dictates that the cost of any recognition event equals the cost of its reciprocal. This symmetry forces a double-entry ledger structure, where every transaction must be balanced by a reciprocal entry, guaranteeing the conservation of informational flux on closed sub-systems.

The cost of a completely degenerate configuration diverges, $J(0^{+})\to \infty$, establishing a finite barrier against vacuous (empty-input) states: a recognition event requires both a transaction and its reciprocal to be non-degenerate.

### 3.2. Cellular Complex and DEC Kinematics

The conservation symmetry $J\left(x\right)=J\left(x^{-1}\right)$ implies closed-chain neutrality [48,49]: any closed chain γ has vanishing net circulation (Appendix B), which in continuum language corresponds to ∇×g=0 (with g=−∇Φ) in the quasi-static sector—the topological prerequisite for a globally defined scalar potential Φ. Guided by the emergent discreteness and this conservation structure, the weak-field potential is represented on a cellular complex using discrete exterior calculus (DEC) [35,36,37]. Local informational constraints are encoded as integer-valued 1-cochains on oriented edges, and the potential is a scalar 0-cochain ϕ on vertices. Under closed-chain neutrality, the 1-cochain field is exact and may be written as w=∇ϕ, enabling a variational formulation whose refinement limit recovers a continuum Poisson constraint. Table 1 collects the principal symbols used throughout the paper.

## 4. Derivation of the Newton–Poisson Baseline

### Discrete-to-Continuum (DEC) Bridge: Poisson as the Baseline Theorem

The instantaneous-closure refinement limit fixes the normalization of the discrete mesh action. Theorem 1 below formalizes this limit via the DEC bridge. The Newtonian weak-field sector fixes this normalization in physical units.

We fix the map from discrete constraint variables to ϕ and ρ using (i) the potential/exactness structure from MP and AX1–AX5, (ii) the quadratic expansion (2), and (iii) standard DEC refinement consistency [35,36,37].

**Theorem** **1** (Construction of the Poisson baseline in the instantaneous-closure limit (conditional on DEC refinement and standard mesh-consistency conditions [35]))**.** *Under assumptions (i)–(iii) and DEC refinement convergence (discrete Laplacian → continuum Laplacian as mesh size →0), the discrete Dirichlet energy converges to its continuum form and stationarity yields a Poisson constraint. Fixing the overall coupling by matching the discrete normalization to Newtonian gravity yields*

(3) $$\nabla^{2}\Phi =4\pi G\;\rho .$$

*Under axioms AX1–AX5, conservation (AX4) yields exactness w=∇ϕ on the cellular complex. The near-equilibrium cost $J\left(1+\varepsilon \right)\approx \frac{1}{2}\varepsilon^{2}$ (Equation (2)) produces discrete action*

$$S\left[\phi \right]\;∼\;\sum_{\mathrm{edges}}J\;\left(\frac{\phi_{j}-\phi_{i}}{\Delta \ell }\right)\;\approx \;\sum_{\mathrm{edges}}\frac{1}{2}{\left(\nabla \phi \right)}^{2}\;\Delta \ell \;\overset{\mathrm{refinement}}{\to }\;\int {\left|\nabla \Phi \right|}^{\;2}\;d^{3}x.$$

*Stationarity δS/δϕ=0 yields the discrete Laplacian equation; DEC refinement convergence [35] gives $\nabla^{2}\Phi =\mathrm{source}$. Matching the normalization to Newtonian phenomenology fixes the source as 4πGρ. Full proof is in Appendix B.*

The gravitational constant *G* and the mesh-to-physical-units proportionality are not predicted by the framework; they are fixed by requiring agreement with observed Newtonian phenomenology (e.g., planetary orbits, laboratory Cavendish experiments). This is standard practice in effective field theory: dimensionful coupling constants are inputs determined by matching to known low-energy physics.

The Newtonian baseline is now fixed: the instantaneous-closure refinement limit reduces the framework to Poisson, and all subsequent source-side modifications are additions to this baseline.

Finite equilibration latency is then introduced: the instantaneous-closure limit idealizes zero-delay constraint refresh; on a cellular complex, updates of edge-local constraints at scale *L*∼1/k must propagate across that scale, and causality bounds the fastest possible refresh rate (Section 5.1.2). Section 5 develops the resulting scale-free, source-side modifications to the effective source in full.

## 5. Information Latency and the Fractional Kernel

Allowing finite equilibration modifies the effective source seen by the weak-field potential while preserving linearity and conservation. Two phenomenological assumptions—Assumption AS1 (scale-free equilibration latency, Section 5.1.1) and Assumption AS2 (causal frequency–wavenumber ansatz, Section 5.1.2)—together yield the quasi-static source-side kernel $w_{\ker}\left(k\right)=1+C{\left(k_{0}/k\right)}^{\alpha }$ (Derived Result 1), the section’s central output.

### 5.1. Finite Equilibration: Scale-Free Latency and Causal Closure (Latency Kernel)

#### 5.1.1. Assumption AS1: Scale-Free Latency and Fractional Memory

**Assumption AS1 (Scale-Free Latency).**

Ledger closure exhibits scale-free latency: unclosed transactions persist as a power-law backlog, with no characteristic timescale governing closure.

This is a phenomenological postulate, not derived from gravitational first principles. Possible physical mechanisms that could generate scale-free latency include (i) coarse-graining over unmodeled hierarchical microscopic degrees of freedom, (ii) coupling to cosmological time evolution in an expanding background, or (iii) emergent scale-invariance from critical-point dynamics. None of these mechanisms are specified in the present effective framework; AS1 is adopted as an empirical ansatz whose consequences can be tested against observations. Alternative latency profiles (e.g., an exponential latency with a characteristic timescale) would yield qualitatively different kernels and provide falsification targets.

Unlike electromagnetism (where retardation is fixed by *c*) or diffusive systems (with material-dependent timescales), gravitational dynamics in cosmology exhibits scale-dependent equilibration: structure formation proceeds hierarchically, with smaller systems virializing earlier than larger ones. If the quasi-static response inherits memory from this hierarchical relaxation, scale-free latency could arise naturally. However, this remains speculative and requires connecting the discrete ledger framework to actual cosmological structure formation, which is beyond the scope of this work.

Scale-free latency induces fractional memory in linear response [50,51,52], conveniently represented by a Riemann–Liouville fractional integral acting on the source. Fractional operators provide a compact representation of heavy-tailed memory and yield clear infrared scaling behavior. No specific microscopic mechanism is assumed in this work, though identifying such a mechanism is an important open question for physical interpretation.

**Proposition** **2** (Fractional-memory effective source (conditional on AS1 and linear response))**.** *Under the scale-free latency hypothesis and linear response about the instantaneous-closure baseline, the effective source acquires a fractional-memory contribution:*

(4) $$\begin{array}{cc} \rho_{\mathrm{eff}}\left(t\right) & =\rho \left(t\right)+C\;\tau_{0}^{-\alpha }\;I^{\alpha }\rho \left(t\right),\\ I^{\alpha }f\left(t\right) & =\frac{1}{\Gamma (\alpha )}\int_{0}^{t}{\left(t-t^{\prime }\right)}^{\alpha -1}\;f\left(t^{\prime }\right)\;dt^{\prime }, \end{array}$$

*Here, $\tau_{0}$ is a reference timescale absorbed into $k_{0}$ via AS2. With α∈(0,1) and dimensionless amplitude C (equivalently a causal multiplier $\propto {\left(i\omega +0^{+}\right)}^{-\alpha }$), Appendix C illustrates a compact bridge to standard fractional-calculus results [50,51].*

**Figure 3:**
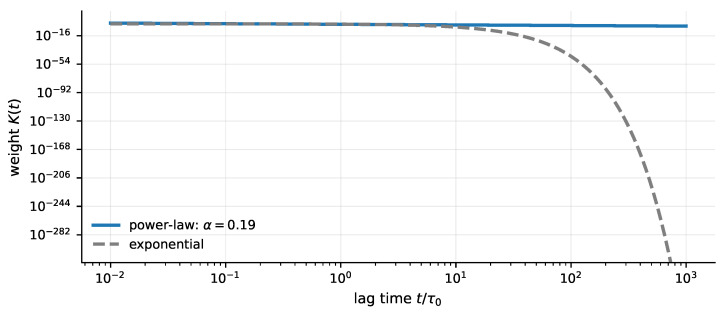
Scale-free latency implies heavy-tailed memory. Under this hypothesis the memory weight is heavy-tailed (a power law), so very old unclosed transactions retain non-negligible influence compared with an exponential (single-timescale) decay. This correspondence is represented by fractional operators in a linear response.

Figure 3 contrasts the scale-free (power-law) memory implied by the scale-free latency hypothesis with a single-timescale exponential for comparison.

#### 5.1.2. Assumption AS2: Causal Closure and the Spatial Modifier

Assumption AS2 assigns each spatial mode *k* an effective refresh frequency $\omega_{\mathrm{eff}}\left(k\right)$ that is consistent with causality and quasi-staticity, and scale-free at a fixed epoch. A causal scaling argument motivates $\omega_{\mathrm{eff}}\left(k\right)\propto k$ under scale-free refresh. In the quasi-static sector, a spatial Fourier mode *k* corresponds to a length scale L∼1/k (or L∼a/k in comoving variables). A “refresh” of the ledger state at scale *L* cannot propagate faster than some finite signal speed $v_{\max}$ (causality). Therefore, the shortest physically allowed refresh time satisfies a ballistic bound

(5) $$\tau_{\mathrm{eff}}\left(k,a\right)≳\frac{L}{v_{\max}}∼\frac{a}{v_{\max}\;k},\;\Rightarrow \;\omega_{\mathrm{eff}}\left(k,a\right)≲\frac{v_{\max}}{a}\;k.$$

Equation (5) establishes a causal ballistic bound: any admissible refresh law must grow no faster than linearly with *k* at a fixed epoch. Imposing scale invariance at fixed *a* rules out new dimensional parameters and selects the minimal power law

(6) $$\omega_{\mathrm{eff}}\left(k,a\right)\propto \frac{k}{a},$$

with the proportionality constant absorbed into $k_{0}$. Alternative scalings—diffusive ($\omega_{\mathrm{eff}}\propto k^{2}/a$) or environment-dependent—are equally consistent with AS1 but yield different spatial kernels. The linear form $\omega_{\mathrm{eff}}\propto k$ is therefore the framework’s primary phenomenological ansatz, not a uniquely forced consequence.

In an expanding background, $\omega_{\mathrm{eff}}\left(k,a\right)∼kc/a$, with order-unity factors absorbed into $k_{0}$. Evaluating the causal multiplier $\propto {\left(i\omega +0^{+}\right)}^{-\alpha }$ from Equation (4) at $\omega =\omega_{\mathrm{eff}}\left(k\right)$ yields the operational Fourier-space kernel form.

The present power-law kernel is therefore conditional on two scaling choices: a heavy-tailed memory law and a ballistic scale-to-refresh mapping. More generally, if the temporal memory law produces a causal multiplier proportional to ${\left(i\omega \right)}^{-\beta }$ and the effective refresh law scales as $\omega_{\mathrm{eff}}\left(k\right)\propto k^{m}$ at a fixed epoch, then the corresponding quasi-static spatial modifier scales as $w_{\ker}\left(k\right)-1\propto k^{-m\beta }$ within the same operator-level bridge. The present construction corresponds to the special case m=1 with β=α. Exponential or stretched-exponential closure laws, or diffusive/environment-dependent refresh maps, would therefore yield different Fourier-space behavior. In that sense, AS1 and AS2 should be read as the defining phenomenological inputs of the scaling window studied here, not as uniquely forced consequences of the ledger scaffold.

**Derived Result** **1.** *Phenomenological spatial kernel (conditional on AS1–AS2 and the* $\omega_{\mathrm{eff}}\propto k$ *ansatz). Under the scale-free latency hypothesis (AS1) and the phenomenological closure ansatz (AS2), evaluating the temporal multiplier at* $\omega_{\mathrm{eff}}\left(k\right)$ *transfers the power law from* ω *to k, yielding*

(7) $$w_{\ker}\left(k\right)=1+C{\left(\frac{k_{0}}{k}\right)}^{\alpha },$$

*and the corresponding source-side modified Poisson relation in the quasi-static weak-field sector,*

(8) $$-k^{2}\Phi \left(k\right)=4\pi G\;w_{\ker}\left(k\right)\;\rho \left(k\right).$$

*Here,* $k_{0}$ *is a conventional reference wavenumber absorbing order-unity factors; equivalently,* $r_{0}:=2\pi /k_{0}$ *is the corresponding real-space transition scale. The kernel parameters are* $\left(C,\alpha ,r_{0}\right)$*, where* α *is the infrared slope,* $r_{0}$ *sets the transition scale, and* C>0 *sets the kernel amplitude. In the strict global-only surrogate used for SPARC fits (Section 6), we denote the fitted surrogate amplitude by A to distinguish it from the theory-amplitude hypothesis C.*

This construction transfers the scale-free temporal memory (AS1) into a scale-free spatial modifier via the $\omega_{\mathrm{eff}}\propto k$ ansatz (AS2), yielding the quasi-static source-side kernel $w_{\ker}\left(k\right)$ that defines the paper’s testable deviation from Poisson (operator-level bridge: Appendix C, Proposition A3). The functional form follows from AS1–AS2 and is not a unique prediction; alternative equilibration mechanisms would yield different kernels.

##### Relation to Common Weak-Field Kernel Parameterizations

As a source-side multiplicative factor in the Poisson closure, $w_{\ker}\left(k\right)$ falls within modified-Poisson/nonlocal-response kernel parameterizations used to test departures from strictly local Newtonian gravity [20,21]. Here, it is restricted to scale-free infrared enhancement with globally shared parameters and conservation-preserving closure, rather than introducing explicit new length scales or environment-dependent interpolation functions.

**Figure 4:**
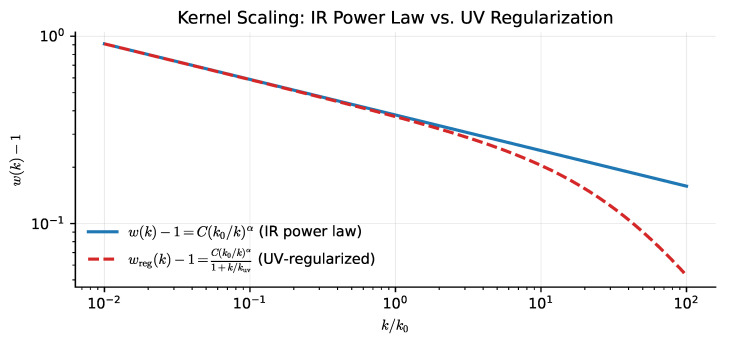
Scale-dependent weak-field response modifier $w_{\ker}\left(k\right)$ under the scale-free latency hypothesis (AS1) and the causal closure ansatz (AS2). The kernel $w_{\ker}\left(k\right)=1+C{\left(k_{0}/k\right)}^{\alpha }$ enhances the effective source at small *k* (large scales, $k\ll k_{0}$) while approaching the Newtonian baseline $w_{\ker}\to 1$ at large *k* (small scales, $k\gg k_{0}$).

Figure 4 shows the resulting scale dependence of the response modifier $w_{\ker}\left(k\right)$ for representative parameters.

The empirical free parameter throughout this work is *A*, fitted to SPARC rotation-curve data in Section 6. The theoretical amplitude *C* and the empirical amplitude *A* are related by source geometry (the surrogate uses a multiplicative ansatz calibrated for disk galaxies); the exponent α and transition scale $r_{0}:=2\pi /k_{0}$ are shared between $w_{\ker}\left(k\right)$ and the empirical surrogate. The quasi-static response is fully characterized by the globally shared triple $\left(A,\alpha ,r_{0}\right)$, with no per-galaxy tuning permitted.

We briefly compare the structural differences between DIF and common alternative phenomenologies.

- Compared with MOND, MOND modifies the force law (acceleration-dependent dynamics) via an interpolation function $\mu (a/a_{0})$ with one global parameter $a_{0}$ [15,16], whereas DIF modifies the effective source via a scale-dependent kernel with three global parameters $\left(A,\alpha ,r_{0}\right)$. On the present SPARC benchmark, MOND is the more economical empirical interpolation, whereas DIF is evaluated here as a tractable causal-response framework for a theory-led retardation kernel. Both are phenomenological; neither is derived from covariant field equations.
- Compared with ΛCDM, ΛCDM adds dark matter halos with per-galaxy profiles (multiple parameters per galaxy) while preserving the standard Poisson response [7], whereas DIF modifies the response law itself but enforces global parameters, making it intermediate in flexibility: more constrained than per-galaxy halos and less constrained than single-parameter MOND.
- Compared with f(R) and other covariant modifications of GR, these theories modify Einstein’s field equations [20], whereas DIF operates only in the non-relativistic quasi-static limit and does not specify a covariant completion, so it cannot currently make predictions for lensing, cosmology, or gravitational waves.
- Compared with generic nonlocal modified-Poisson kernels, which have been studied phenomenologically [21,25], DIF obtains its specific functional form $w_{\ker}\left(k\right)=1+C{\left(k_{0}/k\right)}^{\alpha }$ conditional on phenomenological assumptions AS1–AS2, providing theoretical motivation for the power-law shape within this effective framework, though those assumptions themselves are not derived from deeper principles.

Equations (7) and (8) characterize the quasi-static, linear-response scaling window in which delayed closure can be represented as a scale-free source-side modifier. Outside this window (e.g., fully dynamical regimes, strong-field, or environment-dependent closure mappings), an additional structure would be required and is not assumed here. In particular, no claim is made here that the scale-free power-law form remains valid down to Solar System scales without additional UV regularization or screening.

For the galactic rotation-curve regime studied here, omitted relativistic propagation effects are quantitatively negligible. A light-crossing time across a disk of radius *r*∼10–30 kpc is $\tau_{\mathrm{light}}=r/c\approx 0.03$–0.10 Myr, which is about three orders of magnitude smaller than the fitted memory scale $\tau_{🟉}\approx 133$ Myr and well below a typical orbital timescale $T_{\mathrm{dyn}}$∼200–500 Myr. Thus, $\tau_{\mathrm{light}}\ll \tau_{🟉}\ll T_{\mathrm{dyn}}$ in the SPARC regime, so a fully retarded light-cone treatment reduces to the present quasi-static kernel up to corrections of order $\tau_{\mathrm{light}}/\tau_{🟉}$∼$10^{-3}$. Likewise, for galactic circular speeds *v*∼100–$300\;{\mathrm{km}\;s}^{-1}$, post-Newtonian corrections scale as ${\left(v/c\right)}^{2}$∼$10^{-7}$–$10^{-6}$ and are therefore negligible at current SPARC precision. In a weak-field covariant extension, the temporal kernel would be promoted to a retarded spacetime kernel supported on the past light cone; in the slow-motion limit relevant here, that covariant form would reduce back to the present source-side quasi-static modifier.

A second relativistic check concerns the radial variation in circular speed across the disk. Because the local proper time satisfies $d\tau_{\mathrm{proper}}=dt\sqrt{1-v^{2}\left(r\right)/c^{2}}\approx dt\;\left[1-v^{2}\left(r\right)/\left(2c^{2}\right)\right]$, the effective memory time acquires only a tiny radius-dependent correction, $\delta \tau_{🟉}\left(r\right)/\tau_{🟉}$∼$v^{2}\left(r\right)/\left(2c^{2}\right)$∼$10^{-7}$–$10^{-6}$ over the SPARC velocity range. The dominant radial variation in the response therefore remains classical through the dynamical frequency $\omega_{\mathrm{dyn}}\left(r\right)=v\left(r\right)/r$ already entering the quasi-static scaling window, rather than through special- or general-relativistic clock effects.

## 6. Empirical Consistency Check on SPARC Under Global-Only Constraints

We now ask whether the kernel-induced nonlocal response derived above is compatible with observed galaxy rotation curves under a deliberately strict protocol that limits flexibility. We use the SPARC database of rotation curves as a standardized benchmark and enforce global-only model degrees of freedom across the entire sample: we do not tune parameters per galaxy, we fix the stellar mass-to-light ratio, and we evaluate goodness-of-fit using a conservative total uncertainty model (Appendix D). This is a falsifier-oriented consistency check: non-falsification establishes viability in the galactic regime, not confirmation. Theory-target values for α and *C* are derived independently in Section 7 and can be compared to, but are not required for, the strict global-only evaluation here.

### 6.1. Data, Protocol, and Fit Statistics

#### 6.1.1. Dataset and Strict Global-Only Protocol

We adopt a deterministic analysis subset based on the SPARC galaxy sample quality flag *Q* provided in the galaxy sample table. Following the SPARC convention, we retain galaxies with Q∈{1,2} and exclude galaxies with Q=3 (lower-quality rotation curves). This yields the strict global-only subset used for all SPARC figures and fit statistics reported below, comprising $N_{gal}=147$ galaxies and $N_{tot}=2933$ rotation-curve data points. The quality-flag criterion is applied at the galaxy level (entire rotation curves), and no additional point-level trimming is performed unless stated explicitly.

To minimize hidden flexibility, we impose the following protocol:

- We set M/L=1 globally (no galaxy-by-galaxy adjustment).
- We prohibit per-galaxy tuning: all model parameters are shared across the full sample.
- We evaluate $\chi^{2}$ using a conservative total uncertainty model $\sigma_{\mathrm{tot}}\left(r\right)$ that augments reported measurement errors with floor, beam-smearing, asymmetry, and turbulence terms (Appendix D).

#### 6.1.2. Forward Model: Controlled Surrogate for the Nonlocal Disk Convolution

The kernel modifier $w_{\ker}\left(k\right)$ implies a generally nonlocal mapping from baryonic sources to the gravitational response. A full nonlocal disk convolution can be implemented directly, but it introduces numerical and modeling complexity that is orthogonal to the present falsifier-oriented question. We therefore adopt a controlled surrogate that approximates the kernel action in the quasi-static scaling window as a multiplicative enhancement of the baryon-only rotation curve:

(9) $$v_{\mathrm{model}}^{2}\left(r\right)=v_{\mathrm{baryon}}^{2}\left(r\right)\;\left[1+A{\left(r/r_{0}\right)}^{\alpha }\right],$$

where $v_{\mathrm{baryon}}\left(r\right)$ is the SPARC baryonic template prediction under the fixed M/L protocol and $\left(A,\alpha ,r_{0}\right)$ are global parameters shared by all galaxies.

The parameters $\left(A,\alpha ,r_{0}\right)$ are empirical interface parameters of the surrogate. The theoretical framework motivates the scale-free form and provides a conditional theory-target value for α (Section 7.1), but Equation (9) is not itself the full operator-level prediction for an axisymmetric disk. Rather, it is a controlled empirical interface chosen to test whether a single global scale-free enhancement can survive a deliberately strict no-per-galaxy-tuning benchmark. Accordingly, the surrogate should not be interpreted as a direct measurement of the underlying kernel parameters; a full nonlocal disk convolution may shift amplitudes and objective values even when the infrared slope is preserved.

#### 6.1.3. Goodness-of-Fit Metrics

For each galaxy *g* with $N_{g}$ data points at radii $r_{i}$, observed velocities $v_{obs}\left(r_{i}\right)$, and total uncertainties $\sigma_{\mathrm{tot}}\left(r_{i}\right)$, we compute

(10) $$\chi_{g}^{2}=\sum_{i=1}^{N_{g}}\frac{{\left(v_{\mathrm{obs}}\left(r_{i}\right)-v_{\mathrm{model}}\left(r_{i}\right)\right)}^{2}}{\sigma_{\mathrm{tot}}^{2}\left(r_{i}\right)}\;,\;\frac{\chi_{g}^{2}}{N_{g}}\;(\mathrm{per-galaxy}\;\mathrm{misfit}).$$

In the full sample, the total chi-squared is $\chi_{tot}^{2}=\sum_{g}\chi_{g}^{2}$, and we report a reduced statistic $\chi^{2}/\nu =\chi_{tot}^{2}/\left(N_{tot}-p\right)$ where *p* is the number of global parameters in the model under comparison. We emphasize the distribution of per-galaxy misfit (e.g., $\mathrm{median}(\chi^{2}/N)$) because strict global-only protocols often produce heavy-tailed residuals dominated by a minority of outliers.

### 6.2. Results: Global-Only SPARC Comparison

**Empirical Result** **1.** *Global-parameter SPARC fit (strict global-only protocol). Using the 147-galaxy SPARC sample (Section 6.1; 2933 data points) with three globally shared parameters* $\left(A,\alpha ,r_{0}\right)$*, no per-galaxy tuning, and fixed* M/L=1 *for all galaxies, the surrogate model (Equation (9)) yields*

$$\mathrm{median}\left(\chi^{2}/N\right)=3.06,\;\chi^{2}/\nu =4.63,$$

*with globally shared parameter values*

(11) $$A=0.38,\;\alpha =0.19,\;r_{0}=12\;\mathrm{kpc}.$$

For context under the same strict global-only constraints: a strict global-only ΛCDM/NFW benchmark (two global parameters) yields $\mathrm{median}(\chi^{2}/N)=5.27$, and MOND (one global parameter) yields $\mathrm{median}(\chi^{2}/N)=2.01$. The DIF surrogate is not falsified by rotation curves under a stringent global-only evaluation and outperforms a strict global-only NFW benchmark, while remaining less efficient than MOND under identical constraints. We emphasize, however, that the present DIF benchmark tests a first-pass, theory-led retardation kernel under tight global constraints. In that sense, the benchmark is intended to assess not only fit efficiency but also whether a tractable causal operator framework can survive a deliberately strict global-only evaluation.

### 6.3. Figures and Tables

**Figure 5:**
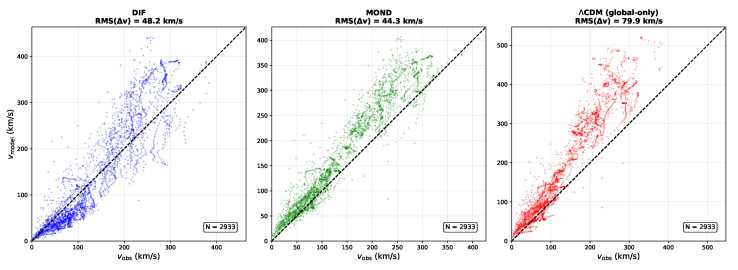
Observed vs. model-predicted rotation velocities for all 147 SPARC galaxies (2933 data points) under strict global-only protocol (fixed M/L=1, no per-galaxy tuning). Each panel shows $v_{\mathrm{obs}}$ vs. $v_{\mathrm{model}}$ scatter for one model: DIF ((**left**), blue), MOND ((**center**), green), and a strict global-only ΛCDM/NFW benchmark ((**right**), red). Dashed line shows 1:1 correspondence.

**Figure 6:**
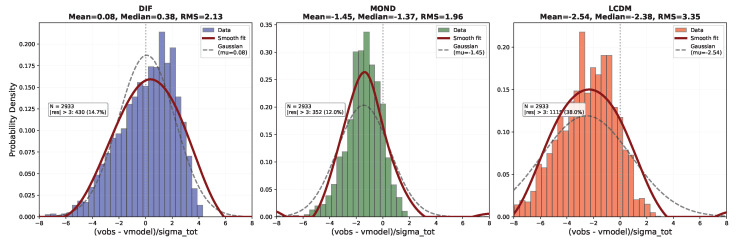
Distribution of normalized residuals $\left(v_{\mathrm{obs}}-v_{\mathrm{model}}\right)/\sigma_{\mathrm{tot}}$ for all 147 SPARC galaxies (2933 data points) under strict global-only protocol (fixed M/L=1, no per-galaxy tuning). Each panel shows one model: DIF ((**left**), blue), MOND ((**center**), green), and a strict global-only ΛCDM/NFW benchmark ((**right**), red). Histogram shows data distribution (colored bars), smooth fitted curve tracing histogram shape (thick red curve, APJ-style spline fit), and a reference Gaussian with the same mean and RMS (black dashed). Gray vertical line marks zero residual. Panel titles report mean, median, and RMS; outlier counts for |residual|>3 quantify extreme deviations.

**Figure 7:**
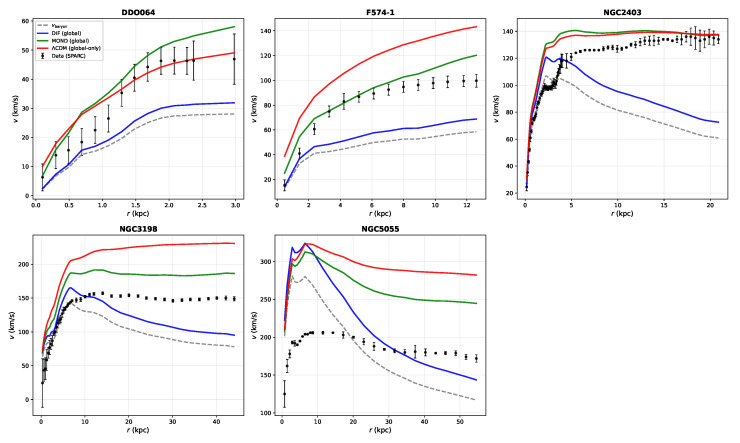
Representative SPARC rotation curves (DDO064, F574-1, NGC2403, NGC3198, NGC5055) under the strict global-only protocol (fixed M/L=1, no per-galaxy tuning). Black points: SPARC data with $v_{\mathrm{err}}$ error bars. Gray dashed: Newtonian baryonic template $v_{\mathrm{baryon}}$. Blue: DIF surrogate (Equation (9), A=0.38, α=0.19, $r_{0}=12\;\mathrm{kpc}$). Green: MOND ($a_{0}=1.2\times 10^{-10}$ m/s^2^, standard ν interpolation). Red: strict global-only ΛCDM/NFW benchmark ($\left(m_{\mathrm{halo}}/m_{🟉},c\right)=\left(30,10\right)$). All three models share parameters globally across the full 147-galaxy sample.

To make the strict global-only consistency check auditable, we include (i) an observed-versus-model scatter plot over the full SPARC sample (Figure 5); (ii) a population-level residual diagnostic (Figure 6); (iii) representative rotation-curve overlays spanning dwarfs to high-mass spirals (Figure 7); and (iv) a benchmark comparison table against MOND and a strict global-only NFW baseline (Table 2).

For the strict global-only ΛCDM benchmark, the ΛCDM curve uses an NFW halo with global concentration *c* and a global halo-to-stellar mass ratio $m_{\mathrm{halo}}/m_{🟉}$; halo masses are assigned via a fixed stellar-mass proxy based on $v_{\mathrm{baryon},\max}^{2}R_{d}$. Standard ΛCDM rotation-curve analyses fit halo parameters per galaxy; this benchmark is intentionally over-constrained to enforce the same global-only policy as DIF and MOND, enabling a fair comparison.

The DIF surrogate sits between MOND and the ΛCDM benchmark (Table 2). Figure 5 shows that the DIF model tracks the 1:1 locus without systematic offset across the full velocity range; Figure 6 shows that normalized residuals are approximately symmetric with mild non-Gaussian tails; Figure 7 confirms that the single set of global parameters traces rotation curves from dwarf irregulars to massive spirals. The non-falsification of a single triple $\left(A,\alpha ,r_{0}\right)$ across 147 galaxies is consistent with a universal scale-free kernel under the adopted surrogate and strict global-only protocol, but it does not by itself establish uniqueness of mechanism or operator-level disk accuracy [17,18]. Relative to MOND, the present underperformance should therefore be interpreted in light of the deliberately constrained, first-pass nature of the kernel used here, which was chosen for tractability and theoretical interpretability rather than for maximal empirical compression.

### 6.4. Interpretation and Falsifiers

Passing a strict global-only rotation-curve test does not confirm the framework, nor does it identify a unique mechanism; it establishes that the kernel-motivated response is not immediately ruled out in the galactic regime under an intentionally conservative protocol.

The present SPARC result is a necessary-but-not-sufficient viability check: it filters out kernel forms that cannot survive global-only rotation-curve scrutiny. Quantitative falsification criteria for the derived parameters α and *C*, globality, and consistency under full disk convolution are collected in Section 7.6. Broader external tests (Solar System PPN, lensing, cluster dynamics) are discussed in Section 9. In particular, a natural next step is to investigate the origin of retardation beyond the present scale-free ansatz, with the aim of refining the kernel while preserving tractability.

## 7. Derived Parameters and Predictions

The preceding sections establish a constrained kernel form $w_{\ker}\left(k\right)=1+C{\left(k_{0}/k\right)}^{\alpha }$ from the discrete variational framework with two dimensionless parameters (C,α) and one-dimensional reference scale $k_{0}$. We now show that the discrete ledger structure determines α uniquely and constrains *C* at the hypothesis level, leaving only the reference wavenumber $k_{0}$ (equivalently the transition radius $r_{0}:=2\pi /k_{0}$) as the single remaining dimensional input.

### 7.1. Derivation of the Kernel Exponent from Self-Similarity

Within the specific additive self-similar closure recursion adopted here, the golden ratio $\phi =(1+\sqrt{5})/2$ is selected as the scaling ratio of the discrete ledger: a geometric scale sequence $1,s,s^{2},\ldots$ closed under additive ledger composition satisfies $1+s=s^{2}$, whose unique positive root is s=ϕ [48,49,53].

The fractional-memory exponent α is determined by a two-scale decomposition argument. Consider a ledger loop (a closed-constraint cycle) at scale ℓφ. Self-similarity and the identity $\varphi^{2}=\varphi +1$ imply that this loop decomposes into two sub-loops at scales *ℓ* and ℓ/φ:

(12) ℓφ=ℓ+ℓ/φ.

This is an algebraic identity (multiply both sides by φ and use $\varphi^{2}=\varphi +1$).

The sub-loop at scale *ℓ* carries a fraction $\ell /\left(\ell \varphi \right)=\varphi^{-1}$ of the parent loop’s scale, so the fraction of closure not completed by this sub-loop is $f_{\mathrm{inc}}:=1-\varphi^{-1}$. We interpret $f_{\mathrm{inc}}$ as the scale-free incomplete-closure fraction associated with the two-scale decomposition.

To connect $f_{\mathrm{inc}}$ to the fractional-memory exponent, we make explicit the (standard) composition rule for fractional orders under serial concatenation in the linear-response regime. In the frequency/Laplace domain, a fractional-memory component of order α contributes a multiplier proportional to ${\left(i\omega \right)}^{-\alpha }$ (equivalently, an operator $\partial_{t}^{-\alpha }$ in time). Two independent serial sub-processes of the same order α multiply their multipliers, yielding an effective order 2α because ${\left(i\omega \right)}^{-\alpha }{\left(i\omega \right)}^{-\alpha }={\left(i\omega \right)}^{-2\alpha }$. In the present two-scale ledger loop decomposition (Equation (12)), the parent loop is represented as the serial closure of exactly two sub-loops, so we equate the effective order to the incomplete-closure fraction:

(13) $$2\alpha \;=\;f_{\mathrm{inc}}\;=\;1-\varphi^{-1}.$$

Solving gives

(14) $$\alpha \;=\;\frac{1-\varphi^{-1}}{2}\;=\;\frac{1}{2}\left(1-\frac{2}{1+\sqrt{5}}\right)\approx 0.191.$$

The derivation uses (i) the closure identity $\varphi^{2}=\varphi +1$, which fixes the two-scale decomposition, and (ii) the additive composition rule for fractional orders under serial concatenation. No adjustable parameters enter once the closure recursion, the two-sub-loop decomposition, and the serial-composition rule are fixed.

**Remark** **1.** *Equation (13) is the functional constraint for α implied by the two-sub-loop closure structure: the factor of 1/2 arises because the decomposition yields exactly two serial sub-loops. If a different decomposition multiplicity n were forced by the closure recursion, the same composition rule would give $n\alpha =f_{\mathrm{inc}}$.*

The appearance of ϕ should therefore be read as a structural consequence of the stated closure recursion, not as an independent empirical input or a standalone observational derivation. If a different self-similar closure rule or a different decomposition multiplicity were forced by the underlying ledger dynamics, a different exponent would result. The rotation-curve comparison in Section 6 is thus presented only as a consistency check for the resulting value α≈0.191, not as an independent derivation of that value from data.

### 7.2. Amplitude Hypothesis

The amplitude *C* enters through the three-channel factorization of the ledger closure across the D=3 spatial dimensions. In this argument, the closure operator in three spatial dimensions factorizes into one longitudinal and two transverse channel contributions; the longitudinal channel carries the full golden-ratio weight $\varphi^{-1}$, while the transverse channels together contribute a complementary factor, yielding the product $C=\varphi^{-1}\cdot \varphi^{-1}=\varphi^{-2}$. The formal argument is developed in the companion framework exposition [49]. The resulting hypothesis is

(15) $$C=\varphi^{-2}\approx 0.382.$$

This value is labeled a hypothesis (not a theorem) because the three-channel factorization relies on additional structural assumptions about the spatial decomposition of the closure operator. The derivation of α in Equation (14) does not depend on *C*.

### 7.3. Predicted Rotation-Curve Enhancement

With the derived exponent and hypothesized amplitude, the kernel is fully specified up to the reference scale $r_{0}$:

(16) $$w_{\ker}\left(k\right)=1+\varphi^{-2}{\left(\frac{k_{0}}{k}\right)}^{(1-\varphi^{-1})/2}.$$

For practical comparison with rotation curves, we relate the Fourier-space kernel to a real-space prediction. For a spherical source, $w_{\ker}\left(k\right)∼k^{-\alpha }$ enhances long-wavelength modes so that the modified enclosed mass scales as $M_{\mathrm{eff}}\left(r\right)\propto r^{1+\alpha }$ at large *r*, giving $v^{2}=GM_{\mathrm{eff}}/r\propto r^{\alpha }$. For disk geometries, the convolution differs in amplitude but the infrared scaling exponent is the same. The theory-predicted surrogate is therefore

(17) $$v^{2}\left(r\right)=v_{\mathrm{baryon}}^{2}\left(r\right)\;\left[1+C{\left(\frac{r}{r_{0}}\right)}^{\alpha }\right],$$

where $v_{\mathrm{baryon}}\left(r\right)$ is the Newtonian baryonic contribution and

(18) $$C=\varphi^{-2}\approx 0.382,\;\alpha =\frac{1}{2}\left(1-\varphi^{-1}\right)\approx 0.191.$$

The only remaining free parameter is $r_{0}$. Note that in the strict global-only empirical test (Section 6), the amplitude is treated as a free empirical parameter *A* (rather than the fixed theory value *C*) to allow the fit to absorb surrogate-approximation errors; comparing the fitted A=0.38 with the predicted C≈0.382 constitutes a non-trivial consistency test.

**Figure 8:**
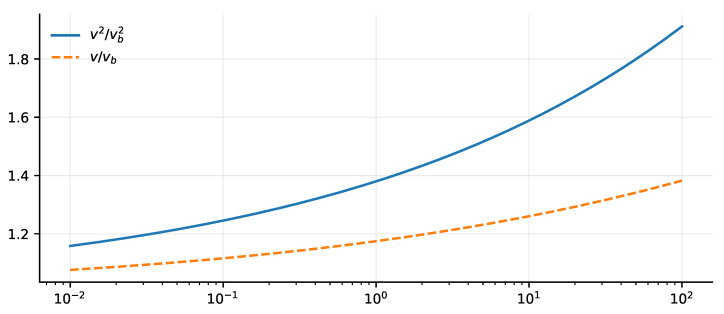
Predicted rotation-curve enhancement from the derived kernel parameters. The enhancement grows as $x^{\alpha }$ in $v^{2}$ (solid) and as $\sqrt{1+C\;x^{\alpha }}$ in *v* (dashed), with $C=\varphi^{-2}\approx 0.382$ and α≈0.191. The transition radius $r_{0}$ is the single remaining dimensional input.

Figure 8 visualizes the predicted scale-free enhancement over the dimensionless radius $x=r/r_{0}$.

### 7.4. Consistency with Galaxy Rotation-Curve Data

The strict global-only SPARC evaluation (Section 6; fixed M/L=1, conservative $\sigma_{\mathrm{tot}}$, Appendix D) yields non-falsification of the framework within the adopted surrogate interface. The conditional theory-target values α≈0.191 and C≈0.382 are numerically close to the empirically fitted surrogate values α=0.19 and A=0.38. This agreement is suggestive, but it should be interpreted cautiously: because the empirical interface is a controlled surrogate rather than the full nonlocal disk operator, the comparison is best read as consistency of the galactic scaling window with the theory-target values, not as a decisive operator-level validation of the underlying kernel. Appendix E further reports sensitivity to the choice of misfit statistic and uncertainty model; those results remain secondary diagnostics of the surrogate-plus-metric combination.

**Figure 9:**
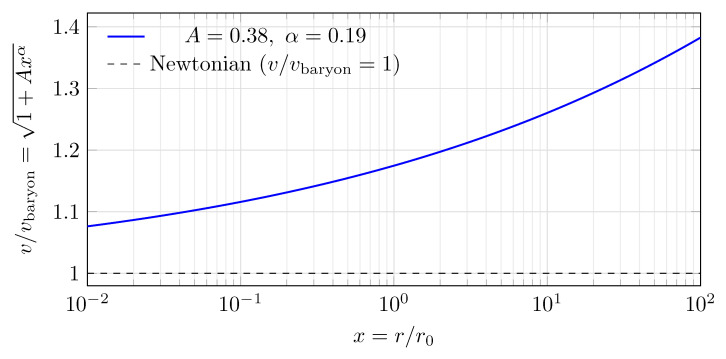
Predicted scale-dependent enhancement of the circular velocity $v/v_{\mathrm{baryon}}=\sqrt{1+C\;x^{\alpha }}$ as a function of $x=r/r_{0}$, evaluated at the derived values $C=\varphi^{-2}\approx 0.382$ and α≈0.191 (Equation (18)).

Figure 9 shows the predicted velocity enhancement evaluated at the derived parameters.

### 7.5. Derivation Status Summary

Table 3 summarizes the epistemic status of each element in the kernel specification.

### 7.6. Falsification Targets

The kernel mechanism with derived parameters is falsifiable through several independent routes:

(i) If future high-resolution rotation-curve analyses yield a best-fit α inconsistent with $(1-\varphi^{-1})/2$ at >3σ, the self-similarity derivation is falsified.
(ii) If the best-fit amplitude is inconsistent with $\varphi^{-2}$ at >3σ, the three-channel hypothesis is falsified (while the exponent derivation may survive).
(iii) If galaxy rotation curves require galaxy-dependent kernel parameters (per-galaxy α or *C*), the global-kernel mechanism is falsified.
(iv) Weak-field lensing observables (once a relativistic completion is specified) or other quasi-static probes sensitive to infrared Poisson modifications provide external falsification routes.

## 8. Limitations and Predictions

### 8.1. Limitations

Several limitations are explicit in the present scope. First, the analysis is non-relativistic, quasi-static, and linear-responsive. For the galactic SPARC regime, this approximation is quantitatively consistent: a light-crossing time across a 10–30 kpc disk is only $\tau_{\mathrm{light}}\approx 0.03$–0.10 Myr, well below the fitted memory scale $\tau_{🟉}\approx 133$ Myr and typical orbital times $T_{\mathrm{dyn}}$∼200–500 Myr, while post-Newtonian corrections scale as ${\left(v/c\right)}^{2}∼10^{-7}$–$10^{-6}$ for galactic circular speeds. The appropriate relativistic completion is therefore not forced by the galactic data themselves, but by the need to extend the framework beyond this well-controlled weak-field scaling window. A viable weak-field covariant embedding would promote the present retarded response to a spacetime kernel coupled to metric potentials, while reproducing the modified Poisson sector derived here in the slow-motion limit.

Second, the scale-free kernel should be interpreted as an effective infrared scaling law rather than a UV-complete prescription. The clean way to formulate the short-distance requirement is to replace the pure power law by a regularized form $w_{\ker}^{\mathrm{UV}}\left(k\right)=1+C{\left(k_{0}/k\right)}^{\alpha }F\left(k/k_{\mathrm{UV}}\right)$, where F(0)=1 preserves the galactic regime and F(x)→0 for x≫1 suppresses short-scale deviations. Such a UV-regularized completion would leave the SPARC-scale behavior essentially unchanged while allowing Solar System/PPN corrections to fall below bounds such as Cassini once the cutoff scale is sufficiently above the galactic transition scale. We do not claim that the form of *F* is uniquely derived here; the point is that Solar System viability should be tested against a UV-regularized extension rather than against naive extrapolation of the unregularized galactic scaling law.

Third, the SPARC comparison uses a controlled multiplicative surrogate (Equation (9)) rather than the full nonlocal disk convolution implied by $w_{\ker}\left(k\right)$. Appendix F now serves as an explicit operator-control check on that choice: for the five representative galaxies examined there, the surrogate tracks the operator-level response at the few-percent curve level, preserves the outer-disk scaling behavior best in the extended spirals, and differs mainly through amplitude renormalization and morphology-dependent details. This does not remove the interest of a future full-sample operator-level refit, but it does show that the main-text interface is a controlled approximation rather than an unconstrained substitution.

Fourth, the kernel construction remains explicitly conditional on phenomenological assumptions AS1–AS2. The exponent α is structurally selected within the specific self-similar closure recursion adopted in Section 7.1, and the amplitude *C* remains a labeled hypothesis rather than a theorem. To make this conditional status concrete rather than merely declarative, the revised kernel discussion now records the nearby scaling family $w_{\ker}\left(k\right)-1\propto k^{-m\beta }$ generated by alternative memory laws and refresh scalings, so the reader can see exactly which part of the result is robust and which part is ansatz-dependent.

### 8.2. Predictions/Falsifiers Subsection

The framework is sharpened by the following falsification targets:

(i) Precision weak-field Solar System constraints, which act as an explicit falsifier of any UV-extended version of the kernel unless additional regularization/screening or a controlled breakdown of the scaling-window assumptions is supplied;
(ii) Lensing observables that compare dynamical and gravitational potentials;
(iii) Reproduction (or failure) of rotation-curve trends when the surrogate interface is replaced by the full nonlocal disk convolution implied by $w_{\ker}\left(k\right)$;
(iv) Cross-system universality: the same globally shared scaling exponent and normalization rules must apply across dwarfs and high-mass spirals under a protocol that prevents per-object tuning.

## 9. Conclusions and Outlook

We have presented a cost-first discrete variational framework. The Recognition Composition Law (RCL) serves as the sole primitive, uniquely selecting a reciprocal closure cost that forces a discrete double-entry ledger of conserved edge-local constraints on a cellular complex. From this emergent structure, the Newton–Poisson equation arises as the instantaneous-closure refinement limit. Finite equilibration introduces a constrained class of source-side modifications characterized by a scale-free response kernel. The construction is linear, conservative, and falsifiable in the non-relativistic, quasi-static regime.

The kernel exponent $\alpha =\frac{1}{2}\left(1-\varphi^{-1}\right)\approx 0.191$ is derived from self-similarity of the discrete ledger closure, and the amplitude $C=\varphi^{-2}\approx 0.382$ is identified as a labeled hypothesis from a three-channel spatial factorization. We evaluate the resulting kernel-motivated response against SPARC galaxy rotation curves under a strict global-only protocol (Section 6; fixed M/L=1, no per-galaxy tuning, conservative $\sigma_{\mathrm{tot}}$). In this deliberately over-constrained setting, the surrogate interface achieves $\mathrm{median}(\chi^{2}/N)=3.06$ across 147 galaxies (2933 points), outperforming a strict global-only NFW benchmark and remaining less efficient than MOND under identical constraints. This result should be interpreted as a galactic-regime viability check of a quasi-static scaling window under a deliberately strict global-only benchmark, not as a full operator-level disk solution, a relativistic theory of gravity, or a demonstration of Solar System compatibility. While MOND remains the more economical empirical interpolation on this benchmark, the present DIF construction is evaluated here as a tractable causal-response framework rather than as a purely empirical scaling law; accordingly, the current underperformance indicates where a better-founded retardation model may be needed. Appendix E records an exploratory re-optimization study intended to quantify sensitivity of globally shared surrogate parameters to the choice of objective function.

At the level of scaling behavior, the modification enters through $w_{\ker}\left(k\right)=1+C{\left(k_{0}/k\right)}^{\alpha }$, i.e., an infrared enhancement that approaches the Newtonian baseline as k→∞. However, the power-law scaling form by itself does not establish Solar System viability under naive real-space extrapolation: inserting the strict-protocol surrogate scaling $\Delta \left(r\right)\equiv A{\left(r/r_{0}\right)}^{\alpha }$ with the fiducial global-only values used in Section 6 (e.g., A≃0.38, $\alpha ≃0.19,r_{0}≃12\;\mathrm{kpc}$) gives $\Delta 1\;\mathrm{AU}∼6\times 10^{-3}$, which is not automatically negligible by Solar System standards. No Solar System compatibility claim is made for the scale-free power-law form without an explicit UV completion and relativistic embedding. For reference, Solar System tracking bounds such as the Cassini constraint on |γ−1| are at the $10^{-5}$ level, so a percent-level modification would generally require additional suppression if it mapped directly onto the PPN sector [54]. A convenient way to state the required suppression is through a UV-regularized completion $w_{\ker}^{\mathrm{UV}}\left(k\right)=1+C{\left(k_{0}/k\right)}^{\alpha }F\left(k/k_{\mathrm{UV}}\right)$ with F(x)→0 at large *x*, which leaves the galactic infrared behavior intact while damping short-scale deviations. Establishing such a completion—together with a relativistic embedding needed to translate the modified source sector to PPN and lensing observables—is beyond the scope of the present quasi-static effective model.

The connection between the kernel parameters and the broader informational framework, including the derivation of dimensional scales from the ledger structure, is a natural direction for future work. The most immediate next falsifiers are a full nonlocal disk-convolution analysis of the SPARC interface and any relativistic embedding capable of translating the kernel to lensing and post-Newtonian observables while remaining compatible with Solar System bounds. Appendix F should be read as a first operator-level control step in that direction rather than as the final numerical word on the disk problem. More specifically, future work should investigate the theoretical basis of retardation beyond the present scale-free latency ansatz as a route to a more empirically competitive kernel while preserving tractability.

Gravitational lensing is not addressed within the present non-relativistic formulation. A relativistic completion would need to specify how the kernel couples to spacetime metric potentials, making lensing an important external constraint on any viable extension of the model.

## Figures and Tables

**Table 1:** Notation summary for the discrete informational framework and its rotation-curve surrogate. Note: the integer 1-cochain *w* (edge-local constraint field) and the Fourier-space kernel modifier $w_{\ker}\left(k\right)$ are distinct objects sharing notation for brevity; context disambiguates.

Symbol	Type	Meaning/Section
*K*	cellular complex	Underlying discretization (Section 3.2)
V,E	sets	Vertices and oriented edges of *K*
ϕ	0 cochain	Scalar potential on vertices
*w*	integer 1-cochain	Edge-local constraint field (exact when neutrality holds)
w(e)	integer	Value of the 1-cochain on edge e∈E
J(x)	cost functional	Reciprocal closure cost fixed by RCL (Section 2)
*x*	positive ratio	Abstract mismatch variable entering *J*
Φ,ρ	fields	Newtonian potential and mass density (Section 4)
$w_{\ker}\left(k\right)$	kernel	Fourier-space response modifier (Section 5.1)
$k_{0}$	scale	Reference wavenumber setting the transition scale in $w_{\ker}\left(k\right)$; absorbed order-unity factors (Section 5.1)
α	exponent	Kernel exponent; derived: $\alpha =\frac{1}{2}\left(1-\varphi^{-1}\right)\approx 0.191$ (Section 5.1 and Section 7.1)
*C*	amplitude	Kernel amplitude; hypothesis: $C=\varphi^{-2}\approx 0.382$ (Section 7.2)
$r_{0}$	scale	Transition radius in kpc; single remaining free (dimensional) parameter (Equation (9))

**Table 2:** SPARC rotation-curve comparison under *strict global-only protocol*: all three models use fixed M/L=1 for all galaxies with no per-galaxy tuning. DIF: 3 global parameters $\left(A,\alpha ,r_{0}\right)$; MOND: 1 global parameter; ΛCDM benchmark: 2 global parameters. Fit quality is summarized by the APJ-style median per-galaxy statistic $\mathrm{median}(\chi_{i}^{2}/N_{i})$ and by the global $\chi^{2}/\nu$ (see text). Under identical constraints, MOND achieves the best fit, DIF is intermediate, and the strict global-only ΛCDM benchmark is worst.

Framework	Parameter Policy	SPARC Fit Summary/Notes
DIF (this work)	Global-only: 3 params $\left(A,\alpha ,r_{0}\right)$ Fixed M/L=1 for all gal.	147 gal., strict global-only (fixed M/L=1): $\mathrm{median}(\chi^{2}/N)=3.06$; $\chi^{2}/\nu =4.63$; A=0.38, α=0.19, $r_{0}=12\;\mathrm{kpc}$
MOND	Global-only: 1 param $a_{0}$ Fixed M/L=1 for all gal.	147 gal., strict global-only (fixed M/L=1): $\mathrm{median}(\chi^{2}/N)=2.01$; $\chi^{2}/\nu =4.09$; RAR: rms 0.057 dex on 175 gal. [46]
ΛCDM NFW	Global-only: 2 params $\left(m_{\mathrm{halo}}/m_{🟉},c\right)$ Fixed M/L=1 for all gal.	147 gal., strict global-only (fixed M/L=1): $\mathrm{median}(\chi^{2}/N)=5.27$; $\chi^{2}/\nu =11.36$; $\left(m_{\mathrm{halo}}/m_{🟉},c\right)=\left(30,10\right)$

**Table 3:** Derivation status of the kernel parameters. The exponent is derived from self-similarity; the amplitude is a labeled hypothesis; only the dimensional reference scale remains free.

Quantity	Value	Status	Source
Kernel form $w_{\ker}\left(k\right)$	$1+C{\left(k_{0}/k\right)}^{\alpha }$	Derived	AS1 + AS2 (Section 5)
Exponent α	$\frac{1}{2}\left(1-\varphi^{-1}\right)\approx 0.191$	Derived	Self-similarity (Section 7.1)
Amplitude *C*	$\varphi^{-2}\approx 0.382$	Hypothesis	3-channel factorization (Section 7.2)
Reference scale $r_{0}$	∼12kpc	Free (dimensional)	Not derived; single remaining input

**Figure A1:**
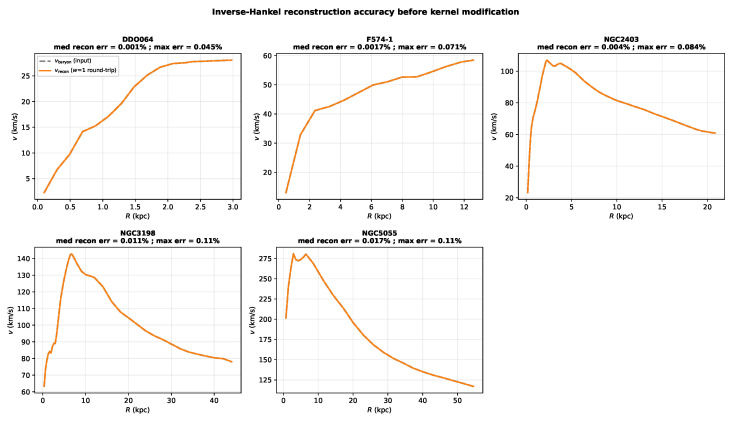
Inverse-Hankel reconstruction accuracy for the five representative SPARC galaxies used in this appendix. Each panel compares the input baryonic velocity profile $v_{\mathrm{baryon}}\left(R\right)$ with the velocity reconstructed from the w=1 (identity kernel) Hankel round-trip, verifying that the numerical pipeline reproduces the Newtonian input to high accuracy before any kernel modification is applied.

**Figure A2:**
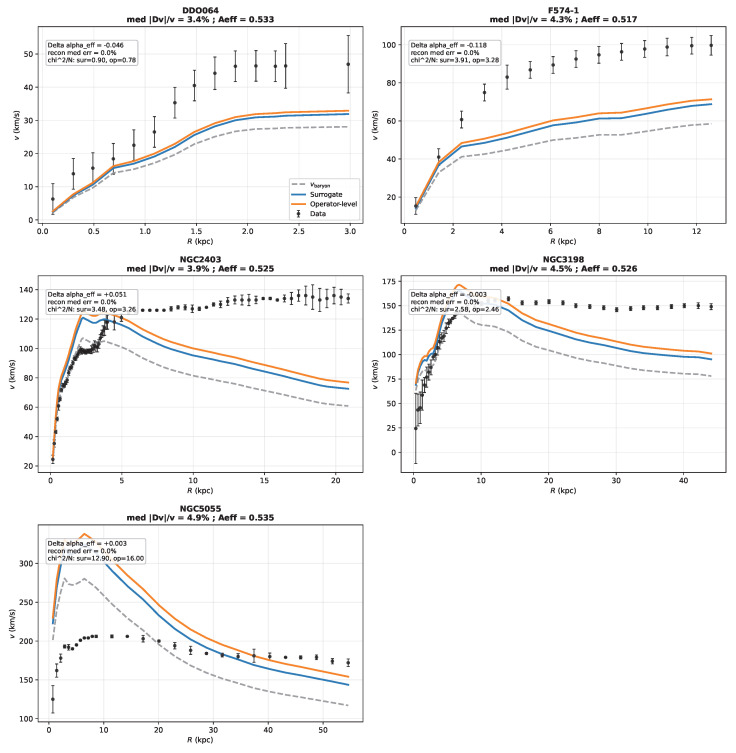
Representative comparison between the multiplicative surrogate used in Section 6 and the operator-level axisymmetric nonlocal response for the five galaxies shown in Figure 7. In each panel, the operator-level curve is obtained by inverse-Hankel reconstruction of the Newtonian baryonic acceleration field and application of the kernel $w_{\ker}\left(k\right)=1+C{\left(k_{0}/k\right)}^{\alpha }$ with C=0.38, α=0.19, and $r_{0}=12\;\mathrm{kpc}$. The surrogate tracks the operator-level curve at the few-percent level in these representative systems, but amplitude-level and morphology-dependent differences remain.

## Data Availability

The publicly available rotation-curve compilation used for empirical comparison is available at https://astroweb.case.edu/SPARC/ (accessed on 16 April 2026) [45]. All derived parameter values reported in this work follow from the analytical expressions in Section 7 with no additional numerical inputs beyond the golden ratio. Figure generation scripts, the operator-level validation pipeline, and all configuration files used to produce the results in this paper are included as Supplementary Material accompanying this submission.

## Supplementary Materials

The following supporting information can be downloaded at: https://www.mdpi.com/article/10.3390/e28040477/s1.

## Appendix A. The *J*(·) Cost Functional and Composition Law

This appendix records the composition-law argument fixing the unique reciprocal quadratic cost used in the main text. The technique of isolating a functional equation and proving uniqueness via regularity and normalization conditions follows the classical approach of Aczél [55]. A complementary “cost-first” ledger framework exposition, including the same reciprocal cost and related structural consequences, appears in [48,49].

### Appendix A.1. Recognition Composition Law (RCL)

**Definition** **A1.**
*Recognition Composition Law (RCL). The cost functional J:(0,∞)→R satisfies the RCL if for all x,y>0,*

(A1)
J(xy)+J(x/y)=2J(x)J(y)+2J(x)+2J(y).

**Proposition** **A1** (RCL forces the canonical reciprocal cost (sketch))**.** *Assume J is a non-constant function satisfying (A1), and is twice differentiable at x=1 with $J^{\prime \prime }\left(1\right)=1$. (Normalization J(1)=0 and reciprocity J(x)=J(1/x) follow as necessary consequences for non-constant J). Then*

(A2) $$J\left(x\right)=\cosh\left(\ln x\right)-1=\frac{1}{2}\left(x+x^{-1}\right)-1.$$

#### Near-Equilibrium Expansion (Used by the DEC Bridge)

The main text requires only the quadratic behavior of *J* near x=1. Using (A2), let x=1+ε with |ε|≪1. Then $\ln\left(1+\varepsilon \right)=\varepsilon +O\left(\varepsilon^{2}\right)$ and $\cosh u-1=\frac{1}{2}u^{2}+O\left(u^{4}\right)$, hence

$$J\left(1+\varepsilon \right)=\frac{1}{2}\;\varepsilon^{2}+O\left(\varepsilon^{3}\right),$$

which is Equation (2) in the main text.

**Remark** **A1.**
*The decoupled quadratic branch (no interaction term) yields $J\left(x\right)\propto {\left(\ln x\right)}^{2}$ and corresponds to an additive, non-interacting composition law. The coupled branch selected by RCL is the minimal genuinely interacting symmetric quadratic law, and it is the branch used throughout this paper.*

### Appendix A.2. Uniqueness Within the Quadratic Symmetric Composition Family

**Proposition** **A2** (D’Alembert constraint: uniqueness of RCL within the quadratic symmetric family (sketch))**.** *Let J:(0,∞)→R be a non-constant continuous function. Assume there exists a symmetric quadratic polynomial $P\left(u,v\right)=au+bv+cuv+du^{2}+ev^{2}+f$ such that for all x,y>0,*

(A3) J(xy)+J(x/y)=P(J(x),J(y)).

*Assuming normalization J(1)=0 and reciprocity J(x)=J(1/x) (which are forced by the canonical RCL (A1)), then:*

(a) *Normalization forces P(0,v)=2v, hence b=2, e=0, f=0.*
(b) *Symmetry P(u,v)=P(v,u) forces a=b=2 and d=e=0.*
(c) *If c=0 (decoupled branch), the induced functional equation admits $J\left(x\right)\propto {\left(\ln x\right)}^{2}$.*
(d) *If c≠0 (coupled branch), rescaling J absorbs c into normalization; imposing $J^{\prime \prime }\left(1\right)=1$ fixes the canonical choice c=2.*

*Thus, within the quadratic symmetric family, the coupled law uniquely takes the RCL form P(u,v)=2u+2v+2uv, as given by Equation (A1).*

**Remark** **A2.**
*This is the sense in which the composition law is treated as “forced” rather than fit: once one restricts to (i) the ratio-composition structure, (ii) debit/credit symmetry, and (iii) the minimal genuinely coupled quadratic family, the RCL is the unique non-trivial choice up to normalization.*

## Appendix B. The Ledger Structural Theorems

This appendix records the core structural consequences of AX1–AX5 used in the continuum bridge narrative. We state them at the level needed for the weak-field limit; stronger sufficient conditions and full formalizations are outside the scope of this work.

**Lemma** **A1** (Atomicity)**.** *From AX1 (non-triviality) and AX5 (minimality), each tick carries exactly one atomic edge-local constraint update.*

This lemma formalizes the tick-level discreteness needed to interpret the ledger dynamics as a sequence of atomic local updates in the later variational construction.

**Lemma** **A2** (Closed-chain neutrality)**.** *From AX4 (conservation), any closed chain γ has vanishing net edge-cochain circulation:*

$$\sum_{e\in \gamma }w\left(e\right)=0.$$

This lemma expresses conservation as vanishing circulation on closed chains, which is the key hypothesis used to pass from edge-local updates to a potential representation.

**Lemma** **A3** (Exactness and potentials)**.** *If circulation vanishes on all cycles, there exists a potential ϕ:V→Z such that w=∇ϕ, unique up to an additive constant per connected component.*

This lemma guarantees the existence of a scalar potential generating the edge cochain, enabling the near-equilibrium action to be written as a Dirichlet energy and connected to Poisson in the refinement limit.

**Remark** **A3.**
*The exactness statement is the discrete analogue of ∇×∇ϕ=0 and is the structural input that allows the near-equilibrium ledger action to be interpreted as a Dirichlet energy in the refinement limit.*

## Appendix C. Fractional-Operator Bridge

This appendix records the operator-level bridge between heavy-tailed closure latency and the fractional-memory form used in the kernel mechanism.

**Proposition** **A3** (Fractional memory from heavy-tailed latency (operator-level))**.** *If closure latency has a scale-free tail $\psi \left(t\right)∼t^{-1-\alpha }$ with 0<α<1, then the linear-response effective source acquires a fractional-memory contribution with causal frequency-space multiplier ${\left(i\omega +0^{+}\right)}^{-\alpha }$. Under a scale-free causal closure $\omega_{\mathrm{eff}}\left(k\right)\propto k$, this induces a spatial Fourier modifier $w_{\ker}\left(k\right)-1\propto k^{-\alpha }$ in the quasi-static scaling window.*

This proposition records the standard operator-level bridge from heavy-tailed latency to a fractional multiplier and shows how the same scaling yields $w_{\ker}\left(k\right)-1\propto k^{-\alpha }$ under the causal $\omega_{\mathrm{eff}}\left(k\right)\propto k$ identification.

## Appendix D. SPARC Error Model and Goodness-of-Fit Statistics

This appendix records the total uncertainty model $\sigma_{\mathrm{tot}}\left(r\right)$ and the goodness-of-fit statistics used in the strict global-only SPARC comparison (Section 6). The intent is to make the reported $\chi^{2}$ values reproducible from SPARC component templates under a protocol that does not invoke per-galaxy tuning.

### Appendix D.1. Data Access and Sample Definition

We use SPARC rotation-curve data and baryonic component templates (disk, bulge, gas) as provided in the public SPARC release. The analysis sample in Section 6 contains $N_{\mathrm{gal}}=147$ galaxies and $N_{\mathrm{tot}}=2933$ data points, and enforces fixed M/L=1 globally (no per-galaxy adjustment). The sample selection used for the strict global-only SPARC evaluation is stated explicitly in Section 6.1. Briefly, we include all Q=1 galaxies, exclude all Q=3 galaxies, and include Q=2 galaxies except for a fixed list of 16 exclusions; this yields $N_{\mathrm{gal}}=147$ and $N_{\mathrm{tot}}=2933$ for all Section 6 figures and misfit statistics.

### Appendix D.2. Total Uncertainty Model

For a data point at radius *r* (kpc) with observed velocity $v_{\mathrm{obs}}\left(r\right)$ (km/s) and reported SPARC uncertainty $v_{\mathrm{err}}\left(r\right)$ (km/s), we define the total uncertainty by adding terms in quadrature:

(A4) $$\sigma_{\mathrm{tot}}^{2}\left(r\right)=v_{\mathrm{err}}{\left(r\right)}^{2}+\sigma_{0}^{2}+{\left(f_{\mathrm{floor}}\;v_{\mathrm{obs}}\left(r\right)\right)}^{2}+\sigma_{\mathrm{beam}}{\left(r\right)}^{2}+{\left(f_{\mathrm{asym}}\;v_{\mathrm{obs}}\left(r\right)\right)}^{2}+\sigma_{\mathrm{turb}}{\left(r\right)}^{2}.$$

We fix the hyperparameters globally for all galaxies as

(A5) $$\begin{array}{cc} \; & \sigma_{0}=10\;\mathrm{km}/s,\;f_{\mathrm{floor}}=0.05,\;\alpha_{\mathrm{beam}}=0.3,\;f_{\mathrm{asym}}^{\mathrm{dwarf}}=0.10,\\ \; & f_{\mathrm{asym}}^{\mathrm{spiral}}=0.05,\;k_{\mathrm{turb}}=0.07,\;p_{\mathrm{turb}}=1.3. \end{array}$$

We classify a system as a “dwarf” if $\max(v_{\mathrm{obs}})<80\;\mathrm{km}/s$; otherwise it is treated as a spiral for the asymmetry term.

#### Appendix D.2.1. Beam-Smearing Term

Let $R_{d}$ be the disk scale radius (kpc) from SPARC metadata; if missing we use $R_{d}=2$ kpc as a default. We define a beam length scale $b=0.3R_{d}$ and set

(A6) $$\sigma_{\mathrm{beam}}\left(r\right)=\alpha_{\mathrm{beam}}\;\frac{b\;v_{\mathrm{obs}}\left(r\right)}{r+b}.$$

#### Appendix D.2.2. Turbulence Term

With the same $R_{d}$, we use

(A7) $$\sigma_{\mathrm{turb}}\left(r\right)=k_{\mathrm{turb}}\;v_{\mathrm{obs}}\left(r\right){\left(1-e^{-r/R_{d}}\right)}^{p_{\mathrm{turb}}}.$$

All terms are added in quadrature and $\sigma_{\mathrm{tot}}\left(r\right)$ is the positive square root of Equation (A4).

### Appendix D.3. Goodness-of-Fit Statistics

For each galaxy *g* with $N_{g}$ points, we compute

(A8) $$\chi_{g}^{2}=\sum_{i=1}^{N_{g}}\frac{{\left(v_{\mathrm{obs}}\left(r_{i}\right)-v_{\mathrm{model}}\left(r_{i}\right)\right)}^{2}}{\sigma_{\mathrm{tot}}^{2}\left(r_{i}\right)},\;\frac{\chi_{g}^{2}}{N_{g}}\;(\mathrm{per-galaxy}\;\mathrm{misfit}).$$

Over the full sample, $\chi_{\mathrm{tot}}^{2}=\sum_{g}\chi_{g}^{2}$ and

(A9) $$\chi^{2}/\nu =\chi_{\mathrm{tot}}^{2}/\left(N_{\mathrm{tot}}-p\right),$$

where *p* is the number of globally shared model parameters (e.g., p=3 for the surrogate DIF interface). We report the distribution of $\chi_{g}^{2}/N_{g}$ (including the median) to diagnose whether fit quality is broad-based or dominated by a small subset of outliers.

## Appendix E. Exploratory Global-Only Re-Optimization Study

This appendix records an exploratory global-only re-optimization analysis intended to quantify the sensitivity of the strict global-only SPARC conclusions (Section 6) to the choice of misfit statistic and (optionally) to modest variations in the uncertainty model. This study is secondary to the main falsifier-oriented benchmark reported in Section 6, and it is included to improve transparency about parameter sensitivity.

### Appendix E.1. Model and Shared-Parameter Constraint

We use the same surrogate interface as in Equation (9),

(A10) $$v_{\mathrm{model}}^{2}\left(r\right)\;=\;v_{\mathrm{bar}}^{2}\left(r\right)\;\left[1+A\;{\left(r/r_{0}\right)}^{\alpha }\right],$$

where $v_{\mathrm{bar}}\left(r\right)$ is the fixed-M/L baryonic template prediction and the parameters $\left(A,\alpha ,r_{0}\right)$ are constrained to be global (shared across all galaxies). No per-galaxy tuning is permitted.

### Appendix E.2. Alternative Objective Functions

Since strict global-only fits can exhibit heavy-tailed residual behavior dominated by a minority of outliers, we consider two complementary global-only objectives:

#### Appendix E.2.1. Objective 1 (Global Reduced Misfit)

Using the same $\sigma_{\mathrm{tot}}\left(r\right)$ definition as in Appendix D, define $\chi_{\mathrm{tot}}^{2}=\sum_{g}\chi_{g}^{2}$ and $\chi^{2}/\nu =\chi_{\mathrm{tot}}^{2}/\left(N_{\mathrm{tot}}-p\right)$ with p=3. The corresponding objective is

(A11) $$L_{1}\left(A,\alpha ,r_{0}\right)\;=\;\chi^{2}/\nu .$$

#### Appendix E.2.2. Objective 2 (Median Per-Galaxy Misfit)

Define the per-galaxy misfit statistic $\chi_{g}^{2}/N_{g}$ as in Equation (A8) (Appendix D), and set

(A12) $$L_{2}\left(A,\alpha ,r_{0}\right)\;=\;{\mathrm{median}}_{g}\;\left(\chi_{g}^{2}/N_{g}\right).$$

This objective down-weights extreme outliers and is often more diagnostic of broad-based performance under global-only constraints.

### Appendix E.3. Optional Uncertainty-Model Sensitivity (If Used)

The primary analysis uses the conservative $\sigma_{\mathrm{tot}}\left(r\right)$ specified in Appendix D. If an alternative uncertainty variant is explored (e.g., removing a single floor term or modestly adjusting a hyperparameter), it should be stated explicitly here and reported as a separate row in Table A1. If no alternative is explored, this subsection may be omitted without loss of completeness.

### Appendix E.4. Parameter Uncertainty Estimation

To summarize sensitivity in a way that does not rely on per-galaxy tuning, we estimate uncertainty on the globally shared $\left(A,\alpha ,r_{0}\right)$ via a galaxy-level resampling procedure:

(i) draw bootstrap resamples of galaxies (sampling galaxies with replacement, keeping each galaxy’s data points intact);
(ii) re-optimize $\left(A,\alpha ,r_{0}\right)$ for each resample under $L_{1}$ and/or $L_{2}$;
(iii) report median and central 68% intervals across resamples as an indicative global-only uncertainty.

This bootstrap is intended as an exploratory sensitivity diagnostic, not a definitive posterior inference.

### Appendix E.5. Results Summary

Table A1 records the exploratory global-only re-optimization outputs under the objectives above. The strict global-only benchmark reported in Section 6 remains the primary headline result of the manuscript.

**Table A1:** Exploratory global-only re-optimization summary for the surrogate model in Equation (A10). Report best-fit values and (if computed) bootstrap 68% intervals under each objective.

Objective	*A*	α	$r_{0}$ (kpc)	Summary Misfit (L)
$L_{1}=\chi^{2}/\nu$	1.6833	1.0000	80.57	3.7111
$L_{2}=\mathrm{median}\left(\chi_{g}^{2}/N_{g}\right)$	1.3878	0.6323	4.28	2.2554

*Note:* Bootstrap intervals were not computed for the values reported in Table A1.

We clarify what the re-optimization does (and does not) imply about α. The globally shared surrogate parameters $\left(A,\alpha ,r_{0}\right)$ in Appendix E are interface parameters for the multiplicative closure ansatz (Equation (A10)), not a direct measurement of the kernel exponent derived in Section 7.1. In particular, allowing the surrogate to optimize under different global-only objectives can drive α toward boundary-seeking values that absorb surrogate mismatch (e.g., heavy-tailed outliers under $\chi^{2}/\nu$) rather than revealing the scaling-window exponent of the underlying nonlocal operator. Accordingly, Appendix E is reported as a sensitivity diagnostic for the surrogate+metric combination; it should not be read as evidence that the SPARC data “prefer” α≈0.19 in the absence of the full nonlocal disk convolution implied by $w_{\ker}\left(k\right)$.

We note that the global $L_{1}=\chi^{2}/\nu$ objective can drive the shared-parameter surrogate to boundary solutions (here, α saturates at the upper bound of the search interval). This behavior is a sensitivity feature of the surrogate interface under a heavy-tailed global objective, rather than a statement about the kernel exponent derived in the theory-target construction. The primary strict-protocol result in Section 6 is therefore reported using a fixed global benchmark, with this appendix included only to document objective-function sensitivity.

### Appendix E.6. Interpretation

The purpose of Appendix E is to make clear how sensitive the shared-parameter surrogate conclusions are to the choice of global-only objective and to modest uncertainty-model variations. Any substantial change in the inferred α or in the relative ranking against benchmarks should be interpreted as evidence that the surrogate interface, rather than the kernel form itself, needs to be replaced by the full nonlocal disk convolution implied by $w_{\ker}\left(k\right)$.

## Appendix F. Representative Operator-Versus-Surrogate Validation Check

This appendix addresses the controlled-surrogate limitation of Equation (9) by comparing the multiplicative SPARC interface used in Section 6 with an axisymmetric operator-level nonlocal response on a representative subset of the exact 147-galaxy included list. The parent sample is the same strict global-only sample used in the main text; the validation subset consists of the five galaxies shown in Figure 7 (DDO064, F574-1, NGC2403, NGC3198, and NGC5055), spanning dwarf through high-mass spiral systems. The purpose is not to claim a full production-level source-density re-analysis of the entire SPARC sample, but to test whether the multiplicative surrogate preserves the scaling-window behavior of the operator-level response closely enough that Section 6 can be read as a first-pass viability check rather than a purely ad hoc fit.

Instead of reconstructing the full source-density files, we work at the level of the Newtonian baryonic radial acceleration field

$$g_{N}\left(R\right)=\frac{v_{\mathrm{baryon}}^{2}\left(R\right)}{R},$$

using the same global M/L=1 baryonic construction as in the main SPARC scripts. For each galaxy, we reconstruct the axisymmetric Hankel/Bessel representation

$$g_{N}\left(R\right)=\int_{0}^{\infty }k\;A\left(k\right)\;J_{1}\left(kR\right)\;dk,\;A\left(k\right)=\int_{0}^{\infty }R\;g_{N}\left(R\right)\;J_{1}\left(kR\right)\;dR,$$

and then apply the same source-side kernel used in the quasi-static construction,

$$w_{\ker}\left(k\right)=1+C{\left(\frac{k_{0}}{k}\right)}^{\alpha },$$

with the same representative parameter values as in Section 6, namely C=0.38, α=0.19, and $r_{0}=12\;\mathrm{kpc}$ (with $k_{0}=2\pi /r_{0}$). The operator-level modified acceleration is then

$$g_{\mathrm{op}}\left(R\right)=\int_{0}^{\infty }k\;w_{\ker}\left(k\right)\;A\left(k\right)\;J_{1}\left(kR\right)\;dk,$$

and the corresponding circular speed is

$$v_{\mathrm{op}}\left(R\right)=\sqrt{R\;g_{\mathrm{op}}\left(R\right)}.$$

We compare this with the surrogate used in the main text,

$$v_{\mathrm{sur}}^{2}\left(R\right)=v_{\mathrm{baryon}}^{2}\left(R\right)\left[1+A{\left(\frac{R}{r_{0}}\right)}^{\alpha }\right],$$

evaluated with the strict-protocol surrogate amplitude A=0.38.

As a numerical sanity check, the inverse-Hankel reconstruction reproduces the input Newtonian baryonic acceleration field to high accuracy before the kernel is applied (Figure A1). Across the five representative galaxies, the median fractional reconstruction error ranges from $1.0\times 10^{-5}$ to $1.7\times 10^{-4}$, and the maximum reconstruction error remains below $1.2\times 10^{-3}$. These values indicate that the numerical differences reported below are dominated by the operator-versus-surrogate mapping rather than by reconstruction failure.

The principal result is that the surrogate and the operator-level response remain close at the curve level for the representative systems studied here (Figure A2). The median fractional velocity difference

$$\frac{|v_{\mathrm{sur}}\left(R\right)-v_{\mathrm{op}}\left(R\right)|}{v_{\mathrm{op}}\left(R\right)}$$

ranges from 3.4% to 4.9% on a per-galaxy basis, with a median across the five systems of 4.3%. The largest pointwise fractional difference encountered among the five systems is 6.8%. Thus, at the level of the circular-speed curves themselves, the multiplicative surrogate tracks the operator-level response reasonably well in the representative cases tested.

To assess whether the surrogate preserves the scaling-window exponent, we fit the enhancement

$$\Delta \left(R\right)\equiv \frac{v^{2}\left(R\right)}{v_{\mathrm{baryon}}^{2}\left(R\right)}-1$$

over the outer-disk comparison window to an effective power law $\Delta \left(R\right)\propto R^{\alpha_{\mathrm{eff}}}$. For the operator-level curves, the fitted values span $\alpha_{\mathrm{eff}}^{\mathrm{op}}=0.072$ to 0.241 across the five representative systems. For the two extended spirals NGC3198 and NGC5055, the fitted operator-level slopes are $\alpha_{\mathrm{eff}}^{\mathrm{op}}=0.187$ and 0.193, respectively, i.e., within ≈0.003 of the surrogate value α=0.19. Larger deviations occur for the more compact systems DDO064, F574-1, and NGC2403. Accordingly, the representative validation supports the use of the surrogate as a scaling-window proxy, but not as a parameter-exact substitute for the operator-level disk response in all morphologies.

Projecting the operator-level enhancement back onto the surrogate form also shows that the amplitude is renormalized relative to the strict-protocol surrogate value. Fitting the operator-level curves to the surrogate form with fixed α=0.19 and $r_{0}=12\;\mathrm{kpc}$ yields effective amplitudes in the range

$$A_{\mathrm{eff}}=0.517–0.535,$$

with median $A_{\mathrm{eff}}=0.526$. Thus, in the representative systems studied here, the multiplicative surrogate captures the shape of the operator-level response better than its absolute normalization: the operator-level mapping is broadly consistent with the same scaling-window trend, but with a moderately larger effective amplitude than the one used in the strict-protocol surrogate benchmark.

For completeness, we also evaluated the same conservative $\sigma_{\mathrm{tot}}$ diagnostic used in the residual analysis of Section 6; the results are collected in Table A2. On the five representative galaxies, replacing the surrogate with the operator-level response modestly improves $\chi^{2}/N$ in four cases (DDO064: 0.90→0.78; F574-1: 3.91→3.28; NGC2403: 3.48→3.26; NGC3198: 2.58→2.46) and worsens it in one case (NGC5055: 12.90→16.00). We therefore do not interpret this appendix as showing uniform fit improvement. Rather, it shows that the main-text surrogate is not grossly misrepresenting the operator-level response in the representative disks examined here, while also confirming that amplitude-level and morphology-dependent differences remain.

**Table A2:** Representative operator-versus-surrogate comparison for the five galaxies used in Figure 7. The operator-level response is obtained by inverse-Hankel reconstruction of the Newtonian baryonic acceleration field and application of the kernel $w_{\ker}\left(k\right)=1+C{\left(k_{0}/k\right)}^{\alpha }$ with C=0.38, α=0.19, and $r_{0}=12\;\mathrm{kpc}$. The reported fractional curve difference is $|v_{\mathrm{sur}}-v_{\mathrm{op}}|/v_{\mathrm{op}}$.

Galaxy	*N*	Median Frac. Diff.	Max Frac. Diff.	$\alpha_{\mathrm{eff}}^{\mathrm{op}}$	$A_{\mathrm{eff}}$	$\chi^{2}/N$ (sur → op)
DDO064	14	0.034	0.049	0.144	0.533	0.90 → 0.78
F574-1	14	0.043	0.053	0.072	0.517	3.91 → 3.28
NGC2403	73	0.039	0.057	0.241	0.525	3.48 → 3.26
NGC3198	43	0.045	0.060	0.187	0.526	2.58 → 2.46
NGC5055	28	0.049	0.068	0.193	0.535	12.90 → 16.00

The correct interpretation of Section 6 is therefore the following. The strict global-only SPARC comparison should be read as a first-pass galactic-regime viability check of the scaling window, carried out with a deliberately simple surrogate interface. This appendix shows that, for representative galaxies, this surrogate reproduces the operator-level axisymmetric response at the few-percent curve level and preserves the outer-disk slope well in the extended spirals, but it is not exact at the level of fitted amplitudes or for all morphologies. A full production-level nonlocal disk analysis based directly on source-density files remains the appropriate next step if one wishes to convert the Section 6 benchmark into a definitive operator-level SPARC test.
